# Polygenic risk score for the prediction of breast cancer is related to lesser terminal duct lobular unit involution of the breast

**DOI:** 10.1038/s41523-020-00184-7

**Published:** 2020-09-07

**Authors:** Clara Bodelon, Hannah Oh, Andriy Derkach, Joshua N. Sampson, Brian L. Sprague, Pamela Vacek, Donald L. Weaver, Shaoqi Fan, Maya Palakal, Daphne Papathomas, Jackie Xiang, Deesha A. Patel, Laura Linville, Susan E. Clare, Daniel W. Visscher, Carolyn Mies, Stephen M. Hewitt, Louise A. Brinton, Anna Maria V. Storniolo, Chunyan He, Stephen J. Chanock, Montserrat Garcia-Closas, Gretchen L. Gierach, Jonine D. Figueroa

**Affiliations:** 1 Division of Cancer Epidemiology and Genetics, National Cancer Institute, Bethesda, MD USA; 2 Division of Health Policy and Management, College of Health Sciences, Korea University, Seoul, Korea; 3 University of Vermont College of Medicine and Vermont Cancer Center, Burlington, VT USA; 4 Department of Surgery, Feinberg School of Medicine, Northwestern University, Chicago, IL USA; 5 Department of Laboratory Medicine and Pathology, Mayo Clinic, Rochester, MN USA; 6 Department of Pathology and Laboratory Medicine, University of Pennsylvania, Philadelphia, PA USA; 7 Laboratory of Pathology, Center for Cancer Research, National Cancer Institute, Bethesda, MD USA; 8 Susan G. Komen Tissue Bank at the Indiana University Simon Cancer Center, Indianapolis, IN USA; 9 Department Internal Medicine, Division of Medical Oncology, College of Medicine, University of Kentucky, Lexington, KY USA; 10 Markey Cancer Center, University of Kentucky, Lexington, KY USA; 11 Usher Institute of Population Health Sciences and Informatics and Cancer Research UK Edinburgh Centre, University of Edinburgh, Edinburgh, UK

**Keywords:** Predictive markers, Breast cancer

## Abstract

Terminal duct lobular units (TDLUs) are the predominant anatomical structures where breast cancers originate. Having lesser degrees of age-related TDLU involution, measured as higher TDLUs counts or more epithelial TDLU substructures (acini), is related to increased breast cancer risk among women with benign breast disease (BBD). We evaluated whether a recently developed polygenic risk score (PRS) based on 313-common variants for breast cancer prediction is related to TDLU involution in the background, normal breast tissue, as this could provide mechanistic clues on the genetic predisposition to breast cancer. Among 1398 women without breast cancer, higher values of the PRS were significantly associated with higher TDLU counts (*P* = 0.004), but not with acini counts (*P* = 0.808), in histologically normal tissue samples from donors and diagnostic BBD biopsies. Mediation analysis indicated that TDLU counts may explain a modest proportion (≤10%) of the association of the 313-variant PRS with breast cancer risk. These findings suggest that TDLU involution might be an intermediate step in the association between common genetic variation and breast cancer risk.

## Introduction

Terminal duct lobular units (TDLUs) are the milk-producing structures of the breast and the predominant anatomical structures from which breast cancers originate^1^. Higher number of TDLUs and higher number of acini (TDLU epithelial substructures) indicate lesser TDLU involution. Among benign breast disease (BBD) patients, having reduced levels of TDLU involution is associated with increased risk of subsequent breast cancer^2,3^. Measures of TDLU involution are thought to reflect a global process occurring throughout the breast^2,4,5^. Two studies from European and Asian populations have also found greater TDLU involution surrounding ER-positive compared with triple negative breast cancers, primarily for acini measures^6,7^. While several hormonal and lifestyle breast cancer risk factors are known to affect TDLU involution^8^, how genetic risk factors relate to TDLU involution is not well understood.

We previously examined whether 62 established breast cancer susceptibility loci were associated with TDLU involution and found limited evidence for an association^9^. Recently, a polygenic risk score (PRS) for the prediction of breast cancer has been derived using 313 common variants from the largest available international genome-wide association dataset^10^. Any observed relationships between the 313-variant PRS and TDLU involution may provide mechanistic clues on the genetic predisposition to breast cancer. ER-specific PRS have also been recently developed^10^. Investigating whether ER-specific PRS are related to measures of TDLU involution could inform whether lobular involution shows differential relationships for genetic susceptibility predisposition to breast cancer subtypes.

Here, we assessed the relation between the 313-variant breast cancer PRS and TDLU involution in 1,398 women with histologically normal donated tissues in the Susan G. Komen Tissue Bank (KTB) at the Indiana University Simon Cancer Center (*n* = 1,089)^11^ and in the background, normal tissue of biopsies among participants diagnosed with BBD in the NCI Breast Radiology Evaluation and Study of Tissues (BREAST) Stamp Project (*n* = 309)^12^.

## Results

### Patients characteristics

Characteristics of the participants are shown in Table 1. The majority of the women were premenopausal (76%), parous (58%), and 22% had a family history of breast cancer in a first degree relative. Approximately 68% of the women had TDLUs observed, with a median of 8.5 TDLUs/100 mm^2^ and 12 acini/TDLU. Age-related TDLU involution metrics were similar in the KTB and the BREAST Stamp studies.

**Table 1:** Descriptive statistics of TDLU involution measures.

Characteristic	Overall (*N* = 1398)	Komen Tissue Bank (*N* = 1089)	BREAST Stamp Project (*N* = 309)
Age (years), *n* (%)			
<30	318 (22.7)	318 (29.2)	0
30–39	234 (16.7)	234 (21.5)	0
40–49	431 (30.8)	274 (25.2)	157 (50.8)
50–59	301 (21.5)	186 (17.1)	115 (37.2)
≥60	114 (8.2)	77 (7.1)	37 (12.0)
Menopausal status, *n* (%)			
Premenopausal	1,053 (76.4)	847 (79.2)	206 (66.7)
Postmenopausal	325 (23.6)	222 (20.8)	103 (33.3)
Parity, *n* (%)			
Nulliparous	581 (41.6)	507 (46.6)	74 (23.9)
Parous	817 (58.4)	582 (53.4)	235 (76.1)
Family history, *n* (%)			
No	1,081 (77.6)	853 (78.3)	228 (75.0)
Yes	312 (22.4)	236 (21.7)	76 (25.0)
Observed TDLUs, *n* (%)			
No	455 (32.5)	369 (33.9)	86 (27.8)
Yes	943 (67.5)	720 (66.1)	223 (72.2)
TDLU count, median (IQR)	3 (0–11)	3 (0–11)	4 (0–14)
TDLU/(100 mm^2^), median (IQR)	8.5 (0.0–31.0)	8.4 (0.0–31.8)	9.7 (0.0–26.7)
Median acini count per TDLU, median (IQR)	12 (7–18.5)	12 (7–19)	11 (7.1–16.5)
Median acini count per TDLU (including women with 0 TDLUs), median (IQR)	7 (0–14.5)	7 (0–15)	8 (0–13.5)
(TDLU counts per unit area) * (acini count/TDLU), median (IQR)	2.4 (0.8–6.4)	2.5 (0.9–7.3)	2.1 (0.6–4.7)
(TDLU count per unit area) * (acini count/TDLU) (including women with 0 TDLUs), median (IQR)	0.8 (0–4.2)	0.8 (0–4.2)	0.9 (0–3.2)

### Association between the polygenic risk score for the prediction of breast cancer and TDLU involution

Greater values of the PRS were statistically significantly associated with higher TDLU counts (*P* = 0.004; Table 2). Specifically, women in the top 25th percentile of polygenic risk had 33% increased odds (95% CI: 1.06–1.65) of having greater TDLU counts compared with women in the bottom 25th percentile. Results were similar, although attenuated, after adjusting for the visually assessed proportion of fibroglandular (nonfatty) tissue. The PRS was not associated with acini counts/TDLU (*P* = 0.808). With respect to the 313 loci that formed the PRS, we found limited evidence for their individual associations with TDLU involution measures (Supplemental Data 1). Analyses stratified by study population suggested a stronger association in the tissue donors (KTB) compared to those diagnosed with BBD (BREAST Stamp) (data not shown). ER-positive and ER-negative PRS were both positively associated with TDLU count (*P* = 0.005 for ER-positive and negative) but not with acini count/TDLU (*P* = 0.861 and *P* = 0.715, respectively).

**Table 2:** Relation of the 313-variant breast cancer polygenic risk score (PRS) with terminal duct lobular unit (TDLU) involution measures obtained in 1,398 women without breast cancer.

	Overall PRS				ER-positive PRS				ER-negative PRS			
	TDLU count		Acini count per TDLU		TDLU count		Acini count per TDLU		TDLU count		Acini count per TDLU	
	RR (95% CI)	*P*	RR (95% CI)	*P*	RR (95% CI)	*P*	RR (95% CI)	*P*	RR (95% CI)	*P*	RR (95% CI)	*P*
PRS continuous	1.12 (1.04, 1.20)	0.004	0.99 (0.92, 1.07)	0.808	1.11 (1.03, 1.20)	0.005	0.99 (0.92, 1.07)	0.861	1.11 (1.03, 1.20)	0.005	0.99 (0.92, 1.06)	0.715
PRS percentile categories												
<25%	1.00	(ref)	1.00	(ref)	1.00	(ref)	1.00	(ref)	1.00	(ref)	1.00	(ref)
25–50%	1.25 (1.00, 1.55)	0.047	1.02 (0.83, 1.27)	0.835	1.30 (1.05, 1.62)	0.018	1.04 (0.84, 1.29)	0.696	1.00 (0.80, 1.25)	0.987	0.87 (0.71, 1.07)	0.166
50–75%	1.31 (1.05, 1.63)	0.017	1.16 (0.95, 1.42)	0.144	1.33 (1.06, 1.66)	0.013	1.19 (0.97, 1.47)	0.104	1.19 (0.95, 1.48)	0.131	0.89 (0.72, 1.10)	0.094
>75%	1.33 (1.06, 1.65)	0.012	0.97 (0.79, 1.19)	0.759	1.37 (1.10, 1.71)	0.006	0.99 (0.81, 1.22)	0.918	1.19 (0.97, 1.48)	0.102	0.92 (0.75, 1.12)	0.311
P-trend	0.011		0.881		0.007		0.736		0.04		0.469	

Poisson models with robust variance adjusted for age and study.

### Mediation analysis

**Fig. 1:**
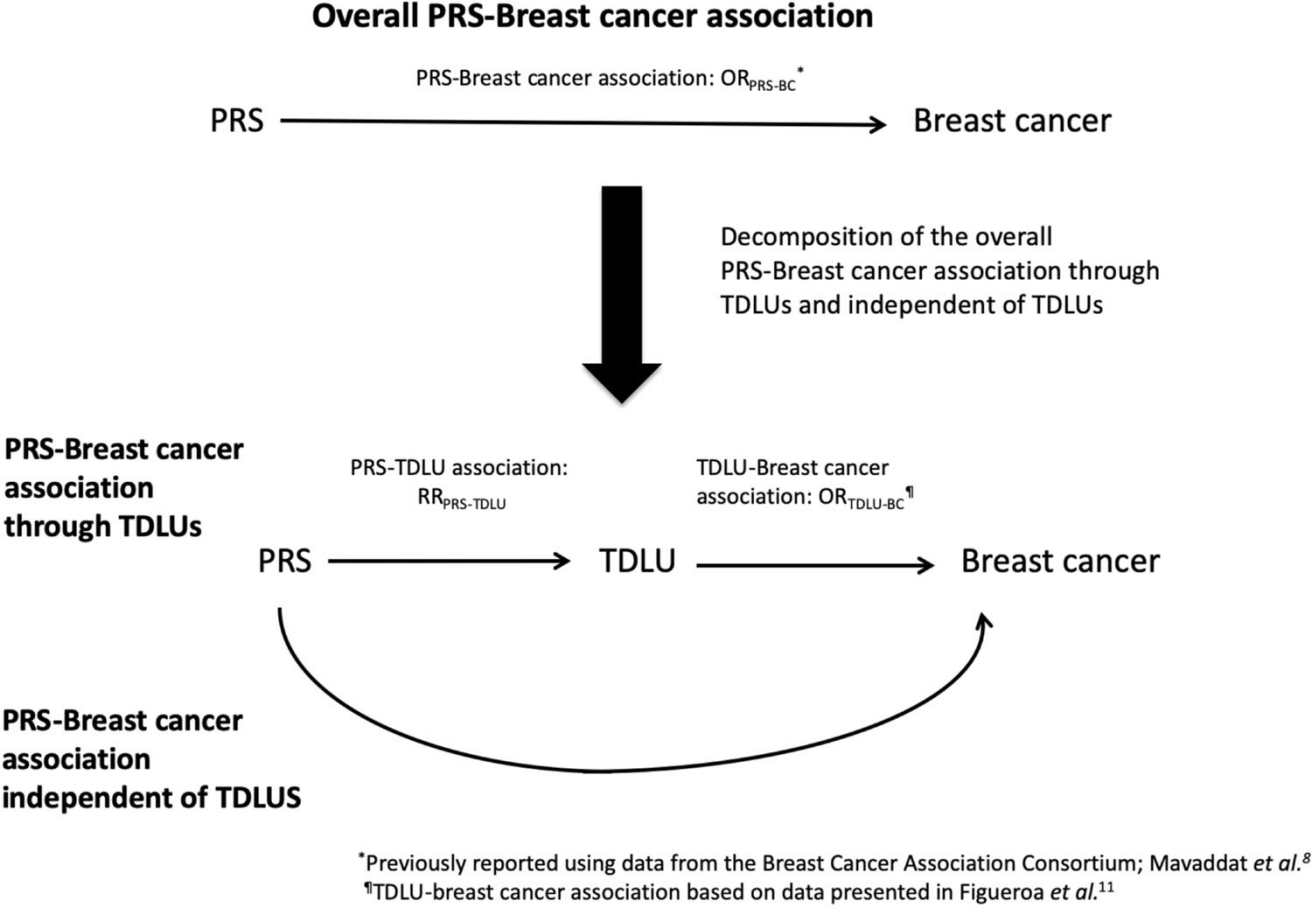
Relationship between the 313-varieant breast cancer polygenic risk score (PRS), terminal duct lobular unit (TDLU) measures and risk of breast cancer. Estimated percent of the association between the 313-variant breast cancer polygenic risk score (PRS) and breast cancer (BC) risk explained by terminal duct lobular unit (TDLU) counts.

Mediation analysis was used to estimate the percent of the association between the PRS and breast cancer risk that might be explained by TDLU count (Fig. 1, Table 3). Ideally such an analysis would be conducted in a study where the PRS, TDLU measures, and breast cancer risk are all available. However, no study, to our knowledge, has all three measures. Therefore, we developed a novel approach where we used information from different sources to estimate the percent explained. The relationship between the PRS and breast cancer risk was obtained from the Breast Cancer Association Consortium^10^; the estimates of the association between TDLU counts (continuous) and breast cancer risk were based on previous results from the Mayo Benign Breast Disease Cohort^13^; and the present analysis provided the relationship between the PRS and TDLU counts. Using this approach, we estimated that ~4 to 7.4% of the relationship between the PRS and breast cancer risk would be explained by TDLU counts, if the odds ratio for the association between TDLU counts and breast cancer risk was between 1.25 and 1.45 based on previous results from the Mayo Benign Breast Disease Cohort.

**Table 3:** Percent of the association between the 313-variant breast cancer polygenic risk score (PRS) and breast cancer risk explained by TDLU counts.

PRS	Breast cancer OR^a,b^	RR^a^	(95% CI)	*P* value	% explained by TDLU counts and 95% CI if OR_TDLU-BC_ = 1.25^c^	% explained by TDLU counts and 95% CI if OR_TDLU-BC_ = 1.35^c^	% explained by TDLU counts and 95% CI if OR_TDLU-BC_ = 1.45^c^
Overall	1.61	1.12	(1.04, 1.20)	0.004	3.97 (1.94, 5.25)	5.75 (2.74, 8.09)	7.41 (3.48, 10.78)
ER+	1.68	1.11	(1.03, 1.20)	0.005	3.47 (1.63, 4.54)	5.05 (2.36, 7.09)	6.52 (2.92, 9.46)
ER−	1.45	1.11	(1.03, 1.20)	0.005	5.28 (2.40, 7.48)	7.49 (3.41, 11.04)	9.54 (4.10, 14.40)

^a^OR and RR per 1 standard deviation change in the PRS.

^b^From the Breast Cancer Association Consortium (Mavaddat et al.^8^).

^c^OR_TDLU-BC_ are continuous TDLU-breast cancer risk estimates based on data presented in Figueroa et al.^11^.

## Discussion

In this study, we assessed the relation between the 313-variant breast cancer PRS and measures of TDLU involution and found that higher values of the PRS were significantly associated with higher TDLU counts in histologically normal/benign breast biopsies. Although individual single nucleotide polymorphisms (SNPs) in the PRS have been associated with some breast cancer risk factors (e.g., mammographic breast density^14^), TDLU involution is the first breast cancer risk factor found to be significantly associated with the 313-variant PRS. Lack of TDLU involution and elevated mammographic breast density are positively correlated histologic and radiologic measures, respectively, of breast tissue composition that are thought to provide independent information about breast cancer risk in women with BBD^15^. Mammographic density was not readily available in the KTB participants, and therefore, we did not have enough statistical power to evaluate the relationship between the 313-PRS and mammographic density in our study. If future studies identify a relation of the 313-PRS with mammographic density, then one could investigate inter-relationships between the PRS, TDLU involution and mammographic density in relationship to breast cancer risk.

Our result relating the 313-PRS with TDLU counts corroborates previous findings showing that family history of breast cancer is associated with increased TDLU counts^8^. Similarly, the lack of observed association between the PRS and acini counts/TDLU is consistent with studies that did not find associations with family history^8^, suggesting that there is not a strong heritable component for the acinar TDLU substructures/size of TDLUs, which may instead be influenced by hormonal/environmental factors (e.g. menopausal hormone use).

Strengths of this study include the well-characterized epidemiologic and genetic data for study participants with standardized, reproducible measures of lobular involution in non-malignant breast tissues. We also applied an innovative analytical approach to estimate the percent of the association between PRS and breast cancer risk potentially explained by TDLU counts. A limitation of the current study is the lack of replication of our findings. Our study is based on observational assessment of TDLU involution. Although we have demonstrated strong within- and between-rater reliability for these TDLU metrics^5,8^, computational pathology approaches may offer future opportunities to advance etiologic studies examining complex relationships between genetic susceptibility, breast tissue composition, and breast cancer risk. In addition, we focused our assessment of involution in the background normal tissue, excluding any observed BBD lobules. Even so, prior work suggests that TDLU involution in histologically normal breast tissue may reflect a global process occurring throughout the breast^2,4,5^. Finally, despite doubling the sample size from our previous analysis^9^, additional samples may be required to detect associations between individual genetic variants and TDLU metrics.

In conclusion, we observed a statistically significant association between a recently developed 313-variant PRS for the prediction of breast cancer and TDLU counts, suggesting that TDLU involution may mediate relationships underlying common genetic susceptibility and breast cancer risk. Future studies may further investigate the use of the PRS for the prediction of TDLU involution, with potential applications in studies that lack measures of lobular involution.

## Methods

### Study population and samples

The study population has been previously described^9^. Briefly, the KTB is an annotated biobank, which has recruited healthy women who provided demographic, lifestyle, and cancer-related information via self-administered questionnaire, blood samples and normal breast tissues. Up to four breast tissue cores were obtained from the upper outer quadrant of the breast using a standardized technique with a 10-gauge vacuum-assisted biopsy. One sample was formalin-fixed and paraffin-embedded and stained with hematoxylin and eosin (H&E). Whole blood samples were collected using Vacuette® EDTA tubes. For this study, a 50 µl aliquot of samples were reconstituted at the Cancer Genomics Research laboratory (Leidos Biomedical Research, Inc., Frederick, MD) for genotyping. Details of the KTB have been described elsewhere (http://komentissuebank.iu.edu/)^11^. The current analysis was restricted to 1507 participants recruited from January 10, 2009 through September 14, 2012, aged 18–91 years, that were eligible for genotyping. Informed consent was obtained from all participants to use of their specimens and questionnaire data for research. Approval for this study was obtained from the Indiana University Institutional Review Board (IRB) and the National Institutes of Health Office of Human Subjects Research (NIH OHSR #4508).

The NCI BREAST Stamp Project is a cross-sectional study of mammographic density conducted among women, aged 40–65 years, who were referred for diagnostic image-guided breast biopsy from 2007 through 2010 at the University of Vermont College of Medicine and University of Vermont Medical Center. Demographic, lifestyle, and breast cancer risk factor information were collected via a self-administered questionnaire and a supplementary telephone interview. Participants underwent clinically-indicated breast biopsies, which were formalin-fixed paraffin-embedded blocks and H&E stained. Whole blood samples and mouthwash samples were collected as previously described^16^. Blood and mouthwash samples were processed at the University of Vermont General Clinical Research Center. Leukocyte DNA was extracted from blood clots using phenol chloroform, and DNA was isolated from buccal cells using Gentra Puregene Buccal Cell Kits (Qiagen). The current analysis was restricted to 450 women who had eligible samples for genotyping. All women provided written informed consent and the study was approved by the IRBs at the University of Vermont and the NCI.

### TDLU involution assessment

H&E slides were scanned for image analysis on Digital Image Hub software (SlidePath/Leica, Dublin, Ireland). The total tissue area (mm^2^) on the slides was outlined using the lasso tool in Digital Image Hub and measured. The study pathologist measured the number of TDLUs (“TDLU count”). Acini count per TDLU was quantified using the TDLU analyzer software^17,18^ as previously described^9^. A high intra-observer agreement (Spearman’s *r* > 0.90) for the TDLU measures was previously reported^5,8^.

### OncoArray genotyping

DNA samples from 1957 women in the two studies (1507 from the KTB and 450 BREAST Stamp project) and 23 quality control samples were genotyped on the Illumina OncoArray chip^19^ at the Cancer Genomics Research laboratory (Frederick, MD, USA). Genotypes were generated using a cluster file provided by the Genetic Associations and Mechanisms in Oncology Consortium. Details of the OncoArray SNP selection and genotyping calling have been described elsewhere19,20. Briefly, ~50% of the SNPs for the OncoArray were selected to provide high coverage for imputation of common variants. The other 50% of SNPs were selected from lists supplied by investigators based on consortia decisions for different diseases. Of the 568,712 variants selected for genotyping on the OncoArray, 533,631 were successfully manufactured on the array (including 778 duplicate probes). Of these 533,631 variants, 38,868 were defined as problematic SNPs by the Consortium QC guideline, thus were excluded from the current analyses.

We further excluded samples and SNPs based on the criteria defined by the consortium QC guideline. Through a stepwise filtration, we excluded SNPs and samples with a call rate <95% and samples with extreme heterozygosity (>0.4 or <0.05) within each study. We also excluded 9 unexpected duplicates with genotype concordance rate >99%, excluded one nonfemale subject with X chromosome heterozygosity close to zero and 66 first-degree relatives based on identity by descent analysis. Using a set of population informative SNPs^20^ and the 1000 Genome phase 3 data, we identified 98 subjects of non-European descent whom we excluded from the analyses.

After genotyping, subjects were further excluded if they had a personal history of in situ or invasive breast cancer, has missing tissue area, pregnant at the time of blood draw, were previous donors (in the KTB), and/or currently taking hormone therapy, leaving an analytic population of 1398 women.

### Imputation

All samples were imputed using the October 2014 (version 3) release of the 1000 Genomes Project dataset as the reference panel and using a two-stage imputation approach using SHAPEIT2^21^ and IMPUTE^22^ version 2, as in Michailidou et al.^23^, run on the NIH High-Performance Computing Biowulf cluster computing (http://hpc.nih.gov). The imputation was done in 5-MB around intervals around each of the 313 loci in the PRS^10^. Imputation quality as assessed by IMPUTE version 2 quality score was >0.80 and *r*^2^ > 0.3 for all loci.

### Statistical analysis

Analyses were conducted combining data from the KTB study and BREAST Stamp Project. The PRS for overall and ER-specific disease was computed using the 313 loci as described in Mavaddat et al.^10^ and the log of effects sizes as weights. We standardized the PRS to have units of standard deviation (SD). With respect to the subtype-specific PRS, we used the weights from the hybrid method in Mavaddat et al., as this method was found to have the best performance.

Similar to our previous analyses^9^, we used Poisson regression models with robust variance to compute per-SD relative risks and 95% confidence intervals for the association of TDLU measures (i.e., TDLU count and acini count per TDLU) with the PRS. The PRS was modeled as a continuous variable and as a categorical variable based on quartiles computed in the entire study population. An offset variable was included in the model to account for the tissue area on the slide. We used the Wald test to assess whether a linear trend between the PRS and morphometric TDLU measures was present. Similar analyses were used to compute per-allele RR (Supplementary Tables 1–2). We also fitted linear regression models to confirm that the associations were not driven by the Poisson model assumptions, similar to our previous analysis^9^, and results were similar. All multivariable models were adjusted for study population (KTB or BREAST Stamp Project) and age. Sensitivity analysis were also carried out by adjusting for family history and visually assessed proportion of fibroglandular tissue. No significant differences were observed. We also conducted stratified analyses by study and by family history (data not shown).

The estimation of the mediating effect was done using the procedure described by Imai et al.^24^ and the 95% CI were obtained by the bootstrap procedure with 10,000 resamples. For this procedure, we assumed that the relationship between the PRS and TDLU could be described by Poisson regression (see above) and that the parameters could be estimated using data from this study. Furthermore, we assumed that the joint effect between PRS and TDLU count on breast cancer could be described by logistic regression Eq. (1):

1 $$\begin{array}{l}P(\rm{Breast}\,cancer = 1|TDLU\,count,\,PRS) = \\ {\mathrm {exp}}(\beta _0 + \beta \times \rm{TDLU}\,count + \gamma \times PRS)/(1 + {\mathrm {exp}}(\beta _0 + \beta \times TDLU\,count + \gamma \times PRS)),\end{array}$$

with parameters estimated so that the three marginal relationships were appropriately constrained (i.e. P(Breast cancer = 1 | PRS) agreed with Mavaddat et al.^10^, P(Brest cancer = 1 | TDLU count) agreed with the assumed value from Figueroa et al.^13^, and f(TDLU | PRS) agreed with results from our study). The constrained model was fit using approaches recently developed by Chatterjee et al.^25^.

All statistical tests were two-sided and *P* values < 0.05 were considered statistically significant. Analyses were conducted using R 3.5.2 on a Mac platform.

### Reporting summary

Further information on research design is available in the Nature Research Reporting Summary linked to this article.

## Supplementary information

Legend Supplemental Data 1

Supplemental Data 1

Reporting Summary Checklist FLAT

## Data Availability

The data generated and analyzed using the KTB specimens will be returned to the KTB and it will available through their virtual repository (https://virtualtissuebank.iu.edu/). To protect patient privacy of the data generated in the STAMP project, de-identified data generated and analyzed will be available on request from Dr. Gretchen L. Gierach (gierachg@mail.nih.gov). The data generated and analysed during this study are described in the following data record: 10.6084/m9.figshare.12293108^26^. The PRS data are in the R file “BC.impute.313.Rdata”. The BREAST Stamp Project data are not publicly available because the informed consent signed by the patients did not include public data sharing. To request these data, contact Dr. Gretchen Gierach (gierachg@mail.nih.gov). However, the Susan G. Komen Tissue Bank (KTB) data are available through the Susan G. Komen Tissue Bank (KTB)’s virtual repository (http://virtualtissuebank.iu.edu) and the phenotype and polygenic risk scores are available from the dbGaP repository under the following accession ID: https://identifiers.org/dbgap:phs002062.v1.p1^27^.

## Code availability

The code developed during the current study are available upon reasonable request.
