# Supplementary material for: Polygenic risk score for the prediction of breast cancer is related to lesser terminal duct lobular unit involution of the breast
Source: NPJ Breast Cancer. 2020 Sep 7;6:41. doi: 10.1038/s41523-020-00184-7 (PMC7477555; doi:10.1038/s41523-020-00184-7)
Supplement: Supplementary file 1 — Legend Supplemental Data 1 [file 41523_2020_184_MOESM1_ESM.pdf]

Supplementary Data. Tables with associations between the 313 loci that formed the PRS TDLU involution measures.
