# Supplementary material for: Polygenic risk score for the prediction of breast cancer is related to lesser terminal duct lobular unit involution of the breast
Source: NPJ Breast Cancer. 2020 Sep 7;6:41. doi: 10.1038/s41523-020-00184-7 (PMC7477555; doi:10.1038/s41523-020-00184-7)
Supplement: Supplementary file 3 — Reporting Summary Checklist FLAT [file 41523_2020_184_MOESM3_ESM.pdf]

nature research

Corresponding author(s): DBPR NPJBCANCER-00524-T

Last updated by author(s): Mar 16, 2020

Reporting Summary

Nature Research wishes to improve the reproducibility of the work that we publish. This form provides structure for consistency and transparency in reporting. For further information on Nature Research policies, see Authors & Referees and the Editorial Policy Checklist.

Statistics

For all statistical analyses, confirm that the following items are present in the figure legend, table legend, main text, or Methods section.

n/a	Confirmed	
☐	☒	The exact sample size (*n*) for each experimental group/condition, given as a discrete number and unit of measurement
☐	☒	A statement on whether measurements were taken from distinct samples or whether the same sample was measured repeatedly
☐	☒	The statistical test(s) used AND whether they are one- or two-sided<br>*Only common tests should be described solely by name; describe more complex techniques in the Methods section.*
☐	☒	A description of all covariates tested
☐	☒	A description of any assumptions or corrections, such as tests of normality and adjustment for multiple comparisons
☐	☒	A full description of the statistical parameters including central tendency (e.g. means) or other basic estimates (e.g. regression coefficient) AND variation (e.g. standard deviation) or associated estimates of uncertainty (e.g. confidence intervals)
☐	☒	For null hypothesis testing, the test statistic (e.g. *F*, *t*, *r*) with confidence intervals, effect sizes, degrees of freedom and *P* value noted<br>*Give P values as exact values whenever suitable.*
☐	☒	For Bayesian analysis, information on the choice of priors and Markov chain Monte Carlo settings
☐	☐	For hierarchical and complex designs, identification of the appropriate level for tests and full reporting of outcomes
☐	☒	Estimates of effect sizes (e.g. Cohen's *d*, Pearson's *r*), indicating how they were calculated

Our web collection on statistics for biologists contains articles on many of the points above.

Software and code

Policy information about availability of computer code

Data collection	Data collection is based on the the Susan G. Komen Tissue Bank (KTB) at the Indiana University Simon Cancer Center , and the NCI Breast Radiology Evaluation and Study of Tissues (BREAST) Stamp Project. Both of these studies have prior publications.
Data analysis	Analyses were conducted using R 3.5.2

For manuscripts utilizing custom algorithms or software that are central to the research but not yet described in published literature, software must be made available to editors/reviewers. We strongly encourage code deposition in a community repository (e.g. GitHub). See the Nature Research guidelines for submitting code & software for further information.

Data

Policy information about availability of data

All manuscripts must include a data availability statement. This statement should provide the following information, where applicable:

- Accession codes, unique identifiers, or web links for publicly available datasets
- A list of figures that have associated raw data
- A description of any restrictions on data availability

The data generated and analyzed using the KTB specimens will be returned to the KTB and it will available through their virtual repository (https://virtualtissuebank.iu.edu/). To protect patient privacy of the data generated in the STAMP project, de-identified data generated and analyzed will be available on request from Dr. Gretchen L. Gierach (gierachg@mail.nih.gov).

Field-specific reporting

Please select the one below that is the best fit for your research. If you are not sure, read the appropriate sections before making your selection.

☒ Life sciences ☐ Behavioural & social sciences ☐ Ecological, evolutionary & environmental sciences

For a reference copy of the document with all sections, see nature.com/documents/nr-reporting-summary-flat.pdf

Life sciences study design

All studies must disclose on these points even when the disclosure is negative.

Sample size	1,398
Data exclusions	Described in methods section of the manuscript.
Replication	No other study with similar measures available. We discuss this in the paper.
Randomization	No relevant. This is an observational study.
Blinding	Not relevant. This is an observational study.

Reporting for specific materials, systems and methods

We require information from authors about some types of materials, experimental systems and methods used in many studies. Here, indicate whether each material, system or method listed is relevant to your study. If you are not sure if a list item applies to your research, read the appropriate section before selecting a response.

Materials & experimental systems

n/a	Involved in the study	
☒	☐	Antibodies
☒	☐	Eukaryotic cell lines
☒	☐	Palaeontology
☒	☐	Animals and other organisms
☐	☒	Human research participants
☒	☐	Clinical data

Methods

n/a	Involved in the study	
☒	☐	ChIP-seq
☒	☐	Flow cytometry
☒	☐	MRI-based neuroimaging

Human research participants

Policy information about studies involving human research participants

Population characteristics	Described in methods section, prior publications and Table 1.
Recruitment	Described in methods section and prior publications.
Ethics oversight	Approval for th e KTB study was obtained from the Indiana University Institutional Review Board (IRB) and the National Institutes of Health Office of Human Subjects Research (NIH OHSR #4508). For the NCI Breast Stamp Project, all women provided written informed consent and the study was approved by the IRBs at the University of Vermont and the NCI.

Note that full information on the approval of the study protocol must also be provided in the manuscript.
